# Isolation, Characterization, and Genome Analysis of a Novel Bacteriophage, *Escherichia* Phage vB_EcoM-4HA13, Representing a New Phage Genus in the Novel Phage Family *Chaseviridae*

**DOI:** 10.3390/v14112356

**Published:** 2022-10-26

**Authors:** Janet T. Lin, Sarah Kirst, Stevan Cucić, Alexandra Klem, Yi-Min She, Andrew M. Kropinski, Hany Anany

**Affiliations:** 1 Guelph Research and Development Centre, Agriculture and Agri-Food Canada, Guelph, ON N1G 5C9, Canada; 2 Department of Molecular and Cellular Biology, College of Biological Science, University of Guelph, Guelph, ON N1G 2W1, Canada; 3 Northern Ontario School of Medicine, Laurentian University, Sudbury, ON P3E 2C6, Canada; 4 Centre for Oncology, Radiopharmaceuticals and Research, Biologic and Radiopharmaceutical Drugs Directorate, Health Canada, Ottawa, ON K1A 0K9, Canada; 5 Department of Pathobiology, Ontario Veterinary College, University of Guelph, Guelph, ON N1G 2W1, Canada; 6 Department of Food Science, University of Guelph, Guelph, ON N1G 2W1, Canada

**Keywords:** bacteriophage, *Escherichia coli*, *Chaseviridae*, STEC O111-specific bacteriophage, genomics, proteomics

## Abstract

Shiga toxin-producing *Escherichia coli* (STEC) is one of the leading causes of foodborne illnesses in North America and can lead to severe symptoms, with increased fatality risk for young children. While *E. coli* O157:H7 remains the dominant STEC serotype associated with foodborne outbreaks, there has been an increasing number of non-O157 STEC outbreaks in recent years. For the food industry, lytic bacteriophages offer an organic, self-limiting alternative to pathogen reduction—one that could replace or reduce the use of chemical and physical food processing methods. From EHEC-enriched sewage, we isolated a novel bacteriophage, vB_EcoM-4HA13 (4HA13). Phenotypic characterizations revealed 4HA13 to possess a myoviral morphotype, with a high specificity to non-motile O111 serotype, and a long latent period (90 min). Through genomic analyses, this 52,401-bp dsDNA phage was found to contain 81 CDS, but no detectable presence of antibiotic resistance, integrase, or virulence genes. A BLASTn search for each of the identified 81 CDS yielded homologues with low levels of similarity. Comparison of RNA polymerase and terminase large subunit amino acid sequences led to the proposal and acceptance of a new bacteriophage family, *Chaseviridae*, with 4HA13 representing a new species and genus. The discovery of this phage has broadened our current knowledge of bacteriophage diversity.

## 1. Introduction

It is unlikely an over-exaggeration that every microbiology laboratory has at least one *Escherichia coli* strain, regardless of their microorganism of study. This bacterium’s speed of replication and robustness of growth are just two of the many traits that have made *E. coli* the darling of the molecular biology world. Ironically, these same traits are partly why their pathogenic brethren remains a continuous source of consternation for safe food production. 

Of the six pathogenic *E. coli* groups, strains capable of producing the Shiga toxin (STEC) are of the most concern to food safety. While cases with less virulent *E. coli* are usually self-limiting, resolving within a matter of days, patients afflicted with STEC can exhibit bloody diarrhea, and 5–10% of cases can be complicated by hemolytic uremic syndrome, with possibility of long-term kidney damage and death [1]. The Shiga toxins (Stx1 and Stx2) target endothelial cells, affecting cell transduction, degrades the ribosome, and induces “ribotoxic stress response” leading to cell apoptosis, mechanisms of which are well-reviewed [2,3,4]. Naturally found in ruminants, STEC is commonly transmitted to humans through the consumption of contaminated water, meats, vegetables, and prepared foods/beverages [5]. However, recent reviews have shown increasing STEC reported in wild and non-ruminant domestic animals and that animal contact has the same exposure risk as undercooked meat [6,7].

From 2015 to 2020, there were a combined 27 national outbreaks investigated by federal agencies in Canada and the US, leading to product recalls and financial loss [8,9]. Within the same timeframe in the United States, there were an additional 157 single-state outbreaks with 3080 illnesses and 293 hospitalizations [5]. O157:H7 is the serotype most have come to associate with *E. coli*-driven foodborne illnesses. However, non-O157 cases have been increasing, largely due to improved laboratory testing and reporting inclusion [10]. Non-O157 STEC cases are largely attributed to one of six serogroups—O26, O45, O103, O111, O121, and O145 [11,12]. As reported by Gould et al. in 2013, the six serotypes combined had almost the same incidence rate as O157. In 2020, CDC reported that non-O157 cases have now increased to 72% of STEC outbreak isolates [13].

As their natural predators, lytic bacteriophages (phages) are good biocontrol candidates for pathogenic microbes. Applications in food safety can either be as a standalone alternative or as part of a hurdle technology to chemical and physical food processing. New STEC phages continue to be isolated and tested in bacterial challenge studies in different food matrices and abiotic surfaces as reviewed by Pinto et al. and Wang et al. [14,15]. O157:H7 remains the primary target, as evidenced by the majority of published STEC phage efficacy studies and the host specificity of PhageGuard EcoShield^TM^—the only phage-based treatment for STEC available commercially. Despite sharing a target host, there can be great diversity between phage candidates. In an effort to better understand *Escherichia* phage diversity, Korf et al. isolated and compared 50 *E. coli* phages [16]. While phenotypic characteristics such as morphotype can be conserved—70% of the phages share a myovirus morphotype—the genotypic characteristics varied greatly, with genomes ranging from 71 to 370 kb of the studied myoviruses. A similar genome size range (40 to 350 kb) was observed in 38 *E. coli* phages isolated from sewage [17]. However, most of them have a siphovirus morphotype. These phages were also spread out across five genera with two phages that were unclassified due to little to no nucleotide similarity to known phages. A recent comprehensive study explored the biology and phage–host interaction of 68 virulent tailed *E. coli* phages [18]. The studied phages belonged to five major *E. coli* phage groups: T-even (subfamilies *Tevenvirinae* and *Vequintavirinae*)-, T1 (family *Drexlerviridae*)-, T5 (family *Demerecviridae* subfamily *Markadamsvirinae*) T3-, and T7 (family *Autographiviridae* subfamily *Studiervirinae*)-related phages. It was found that recognition of host receptors and response to different bacterial immunity systems were associated with the genomic features of the tested phages and determined their host range profiles. Hence, continual isolation and characterization of phages is necessary not only to increase the robustness of phage banks for food safety applications, but also to further our current understanding of phage diversity and provide critical information for the ongoing efforts for a genome-based taxonomical system [19].

In this study, we isolated and characterized the *Escherichia* phage vB_EcoM-4HA13. While kinetic studies suggested this phage to be a less-than-desirable candidate for biocontrol applications, its high specificity can be used to develop a detection assay for *E. coli* O111 strains. Furthermore, comparative genomic analyses showed this phage to be representative of a novel species and genus, and instigated the proposal of a new phage family, *Chaseviridae* [20].

## 2. Materials and Methods

### 2.1. Bacterial Strains and Culturing Conditions

Twenty-two *E. coli* strains were used in this study (Supplementary Table S1). The strains were grown on tryptic soy agar (TSA; BD Canada, Mississauga, ON, Canada) plates at 37 °C for 18–20 h. Liquid cultures were prepared by inoculating 10 mL tryptic soy broth (TSB; BD, Mississauga, ON, Canada) with a single colony and incubated at 37 °C with 180 rpm for 18–20 h. Strains were stored in TSB containing 20% (*v*/*v*) glycerol at −80 °C until further use.

### 2.2. Phage Isolation and Propagation

Raw primary sludge was obtained from the Guelph Wastewater Treatment Plant in Guelph, Ontario, Canada. Fifteen milliliters of the sewage sample were added to an equivalent volume of 2X TSB enriched with a 500 µL cocktail of non-O157 STEC strains (HA2018015 (O111:NM), HA2018016 (O26:H11), HA2018017 (O103:H2), HA2018018 (O145:NM), and HA2018019 (O121:H19)), incubated aerobically at 25 °C with 180 rpm for 18–20 h. After centrifugation at 10,000× *g* for 10 min, the supernatant was filtered using a 0.45 µm cellulose acetate syringe filter (VWR Canada, Mississauga, ON, Canada). The filtrate was spotted, in 10 µL serial dilutions, against each of the enrichment strains on TSA plates with a semi-solid top layer containing 4 mL of tryptic soy broth with 0.5% (*w*/*v*) agar, 200 µL of overnight bacterial culture, and 2 mM CaCl_2_. The plates were incubated at 25 °C for 18–24 h. Prospective plaques were picked and diffused in 500 µL of CM buffer (10 mM MgSO_4_•H_2_O, 6.5 mM CaCl_2_, 6 mM Tris-HCl pH 7.5, 5% (*w*/*v*) gelatin) for a minimum of 3 h at room temperature, and re-spotted against the same bacterial strain. This purification process was repeated three times before propagation.

4HA13 was propagated using an optimized solid agar approach. Phage lysate, overnight bacterial culture, and 2 M CaCl_2_ were combined in a 20:10:1 (*v*/*v*) ratio, and incubated at 25 °C for 15 min to allow for phage adsorption. Ten milliliters of TSB with 0.5% (*w*/*v*) agar and 300 µL of the phage-bacteria-CaCl_2_ mixture were added to large 150 mm TSA plates. After solidification, the plates were incubated at 25 °C for 18–24 h. Ten milliliters of CM buffer were added to each plate and left for diffusion for at least 3 h at room temperature prior to collection and centrifugation at 10,000× *g* for 10 min. The pooled supernatant was filtered using a 0.45 µm cellulose acetate bottle filtration system (VWR Canada) and titred prior to downstream applications.

### 2.3. Phage Host Range Determination

#### 2.3.1. Turbidimetric Assay

Turbidimetric phage host range determination against 22 *E. coli* strains using a microtitre plate was carried out by diluting phage lysate and bacteria with TSB supplemented with 2 mM CaCl_2_ to a final concentration of 10^7^ particles/mL. Equal volumes of the diluted phage and bacteria were added to a 384-well flat-bottom microtitre plate (Corning Scientific, Corning, NY, USA), in triplicates, for an input ratio (phage/bacteria) of 1. Three sets of controls, in triplicates, were included: (1) blank (TSB with 2 mM CaCl_2_), (2) sterility (diluted phage and TSB with 2 mM CaCl_2_), and (3) growth (diluted bacteria and TSB with 2 mM CaCl_2_). The optical density (OD_600_) of each well was read every 30 min for 24 h using a microplate reader (Agilent Technologies, Santa Clara, CA, USA), with continuous orbital shaking (180 cpm) at 25 °C. Sample readings were standardized using the average of the blank controls and analyzed using the Phage–Host Interaction Data Analyzer (PHIDA), which automatically assigns a phage–host interaction designation based on the length of bacteria growth delay and the end-point optical density [21].

#### 2.3.2. Efficiency of Plating (EOP)

EOP was determined by spotting 10 µL serial dilutions of the phage lysate against 22 *E. coli* strains on TSA plates with a semi-solid top layer. The plates were incubated at 25 °C for 18–24 h and the resultant plaque counts were compared with the reference titre of the phage on its propagation host.

### 2.4. Virulence Index Determination

Phage virulence was quantified using the methodology established by Storms et al. scaled down to a total volume of 100 µL and an adjusted input ratio range of 10^−6^ to 10^0^. The assay was performed, in duplicates, in TSB at 25 °C for a duration of 16 h. Optical density values were read by a microplate reader (Agilent Technologies). Determination of MV_50_ and virulence index were determined as described in [22].

### 2.5. One-Step Growth Curve and Adsorption Assay

The one-step growth curve and adsorption assay were performed in triplicates, and in accordance with Kropinski with modifications [23,24]. Free phages were quantified by spotting 10 µL serial dilutions against the propagation host as described above.

### 2.6. Transmission Emission Microscopy

A milliliter of phage lysate was pelleted in a tabletop centrifuge at max speed (25,000× *g*) at 4 °C for 1 h. The pellet was washed with 1 mM HEPES twice before resuspension in 30 µL of 1 mM HEPES and allowed to diffuse at 4 °C overnight. Phage particles were affixed to carbonized copper grids (150 square mesh) and excess liquid was removed with filter paper. The grids were then stained with 2% (*w*/*v*) uranyl-acetate before imaging with a Tecnai F20 (Thermo Scientific, Waltham, MA, USA) transmission electron microscope coupled with a Gatan 4k CCD camera. Phage dimensions were measured using ImageJ and average values were obtained from a minimum of 10 images [25].

### 2.7. DNA Extraction and Genome Sequencing

Genomic DNA was extracted using a PureLink Viral DNA/RNA Mini Kit (Thermo Scientific) per the manufacturer’s protocol. All kit reagents, except for the carrier tRNA, were scaled up 5–10-fold to accommodate the lower phage titre. DNA was eluted in molecular-grade water and quantified with the Qubit dsDNA BR assay kit (Thermo Scientific) and the Qubit 2.0 fluorometer (Thermo Scientific). Its quality was assessed with a Nanodrop spectrophotometer (Thermo Scientific) prior to library preparation and sequencing at the Ottawa Research and Development Centre. DNA was normalized to 200 ng and mechanically sheared using Covaris M220 (Covaris, Woburn, MA, USA). An NGS library was prepared using the NexSeq AmpFREE Low DNA Library Kit (LCG, Lucigen, Teddington, UK), and pooled with other libraries before sequenced with the MiSeq sequencer (Illumina, San Diego, CA, USA) using 2 × 300 bp MiSeq Reagent Kit v3 (600-cycles). All kits were used per their manufacturers’ protocol.

### 2.8. Genome Analyses

#### 2.8.1. Genome Assembly and Annotation

The genome was de novo assembled using the DNASTAR Lasergene suite version 16 (Madison, WI, USA) using a base of 200,000 reads. Contigs were polished and re-assembled to produce a draft genome, which was then re-aligned to a homologue, *Escherichia* phage Mangalitsa (GenBank Accession: MN045229) using progressiveMauve [26].

Genome annotation was performed using a combination of RAST, CPT PAP Structural Workflow v2021.02, and CPT PAP Functional Workflow v2021.01 [27,28]. Identified transmembrane domains were confirmed with TMHMM 2.0 and Phobius [29,30]. The 4HA13 genome and its sequence similarity with closest homologs were visualized with BRIG 0.95 [31].

#### 2.8.2. In Silico Analyses

Phage termini of 4HA13 was determined using PhageTerm 1.0.11 [32]. The presence of antibiotic resistance, virulence, and integrase genes was assessed using ResFinder 4.1, VirulenceFinder 2.0, and ICEfinder, respectively [33,34,35]. 

A pairwise similarities heat map of 4HA13 and its top 49 BLASTn hits from NCBI were generated by VIRIDIC [36,37,38]. Entries of partial or metagenome-assembled genome (MAG) were excluded. A phylogenetic tree was constructed using PATRIC’s Codon Tree, set to 10 amino acid and nucleotide sequences for alignment, and a maximum omission of three genomes [39].

### 2.9. Proteome Analyses

#### 2.9.1. CsCl Purification

A liter of filtered phage lysate (10^8^–10^9^ PFU/mL) was treated with 10 µg/mL of DNase I and RNase A (Sigma Aldrich Canada, Oakville, ON, Canada) and incubated at room temperature with gentle shaking for 1 h. After centrifugation at 7000× *g* at 4 °C for 18 h, the pellets were resuspended in 5–10 mL SM buffer (100 mM NaCl, 8 mM MgSO_4_•H_2_O, 50 mM 1 M Tris-Cl pH 7.5) at 4 °C for 3–4 h. The collected SM buffer was centrifuged at 12,000× *g* at 4 °C for 10 min and the supernatant was transferred to a fresh conical tube. Cesium chloride was gradually added to the supernatant, in four portions, until the final density of the mixture reached 0.817 g/mL. The phage-CsCl mixture was centrifuged in OptiSeal tubes (Beckman-Coulter Canada, Mississauga, ON, Canada) at 155,000× *g* at 4 °C for 24 h (90Ti rotor, Beckman-Coulter). The resultant visible band was extracted using a 20 g needle and syringe (BD Canada) and added to a fresh preparation of CsCl in SM buffer. The centrifugation and extraction process was repeated prior to an overnight dialysis using 3500 MWCO Slide-A-Lyzer dialysis cassettes (Thermo Scientific) against 1% (*w*/*v*) ammonium bicarbonate at 4 °C. Dialysis was repeated.

#### 2.9.2. Liquid Chromatography–Mass Spectrometry

Proteins from 4HA13 were reduced with 10 mM dithiothreitol (DTT), alkylated with 55 mM iodoacetamide, dialyzed against 10 mM ammonium bicarbonate, and dried using a CentriVap centrifugal concentrator (Labconco, Kansas City, MO, USA). The tryptic digestion of proteins was performed using sequencing-grade trypsin (Promega, Madison, WI, USA) in 25 mM ammonium bicarbonate at a 1:100 ratio of trypsin-to-protein substrates. The resulting peptides were dried and reconstituted with 0.2% formic acid (FA), and identified using an online ACQUITY ultra performance liquid chromatography (UPLC) M-class (Waters, Milford, MA, USA) coupled with an Orbitrap Fusion mass spectrometer (MS) (Thermo Scientific). The peptides were trapped by a NanoEase *m/z* symmetry C18 trap column (100 Å, 5 µm, 180 µm I.D. × 20 mm length) for 3 min at the flow rate of 5 mL/min using Solvent A (0.1% FA) at 300 µL/min, and separated on a NanoEase *m/z* HSS C18 T3 analytical column (100 Å, 1.8 µm, 75 µm I.D. × 150 mm length, Waters) at the flow rate of 300 nL/min for 90 min. A linear gradient from 2% to 30% of Solvent B (0.1% FA, 99.9% acetonitrile, Waters) at the duration of 65 min was used for peptide elution, followed by flushing with 85% solvent B for 15 min and re-equilibrating the column with solvent A for 10 min. An MS survey scan was acquired with a high resolution of 120,000 at the mass region of *m/z* 350 to 1800, and MS/MS measurements were performed on multi-charged ions of 2+ to 7+ using low-energy collision-induced dissociation at data-dependent acquisition mode. Dynamic exclusion was set to 30 s. 

The raw LC MS/MS data were searched against the 4HA13 protein sequences using the Mascot Server version 2.6.0 [40]. The search parameters were restricted to tryptic peptides at a maximum of one missed cleavage. Cysteine carbamidomethylation was designated as a fixed modification. Loss of methionine or acetylation at the N-termini, oxidation of methionine, deamidation of asparagine and glutamine, and acetylation of lysine were considered as variable modifications. Mass tolerances were set up to 10 ppm for Orbitrap MS ions and 0.8 D for ion-trap MS/MS fragment ions. Peptide assignments were filtered by the significance threshold *p* value < 0.05, and the identified MS/MS spectra were verified manually.

## 3. Results and Discussion

### 3.1. Biological Characterizations

#### 3.1.1. Morphology

**Figure 1:**
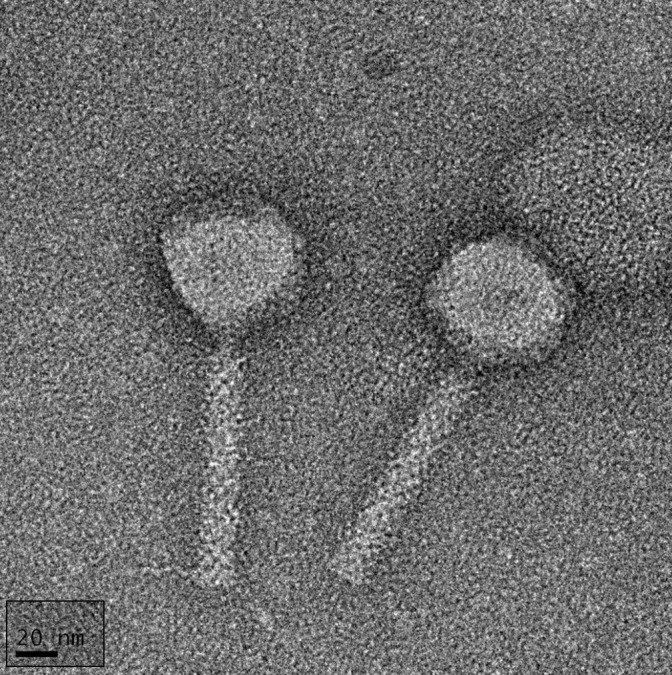
Transmission electron micrograph of 4HA13. Phage particles show the icosahedral head and uncontracted tail. Also visible are the striations from the tail tube proteins and some tail fibers. The image was altered to include the scale bar in the same orientation as the phage particles. Scale bar = 20 nm.

Based on TEM micrographs, vB_EcoM-4HA13 has a myoviral morphotype with a 53.2 ± 2.6 nm wide isometric capsid (Figure 1). Its tail is 11.9 ± 1.6 nm wide and 116.5 ± 2.5 nm long when uncontracted. 

#### 3.1.2. Host Range

Of the 22 *E. coli* strains tested, 4HA13 demonstrated a host specificity for non-motile O111 (O111:NM); in liquid assay, detection was delayed for 13 h (Supplementary Table S1). For motile O111 (O111:H8), 4HA13 reduced the strain’s 24 h growth by 82% but had no effect on detection time. Compared to non-motile O111, 4HA13 against O111:H8 had a very reduced efficiency of plating (EOP) of 1%. There was no effect observed against the other STEC serovars (O26, O45, O103, O121, O145, and O157) or the AMR *E. coli* strains, in both liquid and solid media. In the laboratory strain DH5-α, only a short detection delay of 1–2 h was observed.

#### 3.1.3. Virulence Index of 4HA13 against *E. coli* O111:NM

**Figure 2:**
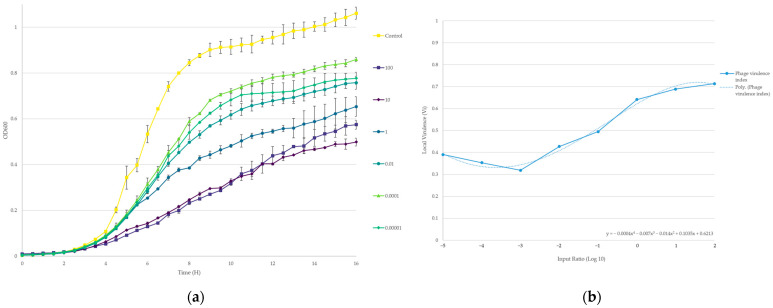
Determination of 4HA13 virulence against *E. coli* O111:NM in TSB, 25 °C. (**a**) Growth of *E. coli* O111:NM treated with 4HA13 at phage-to-host ratios of 10^−5^ to 10^2^ over 16 h. The curves were normalized against an assay blank average; (**b**) local virulence (Vi) values, as determined using the calculations set out by Storms et al., were plotted against the input ratios [22]. A curve of best fit and its polynomial equation allowed for the determination of 4HA13’s virulence index (y = Vi = 0.5) and are indicated by the red dash line.

At input ratios of 10^−5^ to 10^1^, 4HA13 caused a short delay in *E. coli* O111:NM detection at OD_600_ = 0.2 of approximately one hour (Figure 2). Higher ratios of 10^1^ and 10^2^ showed a longer detection delay of approximately 3 h, suggesting a possible threshold-based correlation between phage/host ratio and detection delay. From the growth curves of *E. coli* O111:NM exposed to different amounts of 4HA13, we determined that 4HA13 required an input ratio of 0.16 to reach 50% of its maximum virulence (i.e., kill half of its host population).

#### 3.1.4. Kinetics

**Figure 3:**
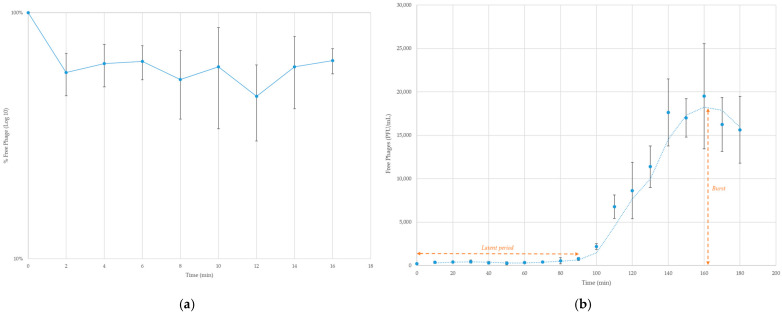
Phage kinetics of 4HA13. (**a**) Adsorption of 4HA13 to *E. coli* O111:NM over 16 min; (**b**) one-step growth curve of 4HA13 to *E. coli* O111:NM. The latent period and burst are marked by orange dash lines.

Compared with some of the other recently published STEC phages, 4HA13 adsorbs poorly to its host, *E. coli* O111:NM [41,42,43,44]. After two minutes, only half (57%) of the phage was adsorbed, and remained unchanged for 16 min (Figure 3a). A statistical t-test confirmed that the observed reduction of free phages between t = 0 and t = 2 is significant (*p* < 0.001). The finding of residual 4HA13 phage particles that had a slower adsorption rate supports previously reported biphasic adsorption kinetics and population heterogeneity in other phages [45,46,47,48]. The observed heterogeneity in adsorption kinetics of T4 phage population was suggested to be a result of two point mutations in long-tail fiber-encoding genes [49]. The low level of adsorption may be a contributing factor in the long latent period (90 min) observed in its one-step growth curve (Figure 3b). On average, recently characterized STEC phages have a latent period of 15 min, with a burst size of 93 PFU/cell [41,42,43,44]. 4HA13 has a burst size of 55 ± 10 PFU/cell.

### 3.2. Genome Characterizations

#### 3.2.1. General Features

**Figure 4:**
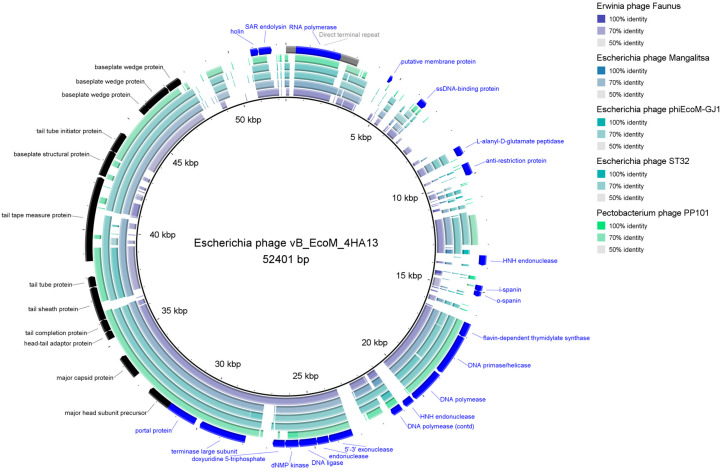
Genome map of 4HA13 and its five closest nucleotide homologues. CDS with annotated functions are highlighted, with structural proteins in black and orientation denoted by arrow-ends. The direct terminal repeat region (3120 bp) is highlighted in grey. The intensity of color of the homologues corresponds to level of nucleotide similarity.

4HA13 has a linear dsDNA genome length of 52,401 bp with a GC content of 42.8% (Accession: MN136198.2, Figure 4). It is predicted to be circularly permutated, with a direct terminal repeat region of 3120 bp. A combination of RAST and CPT PAP Structural Workflow identified 81 coding regions, all located on the sense strand, with no tRNA-encoding genes being present. Of the coding regions, more than half were hypothetical proteins. Notable protein functions identified include nucleotide metabolism (RNA polymerase, endonuclease and exonuclease, DNA primase/helicase, DNA polymerase, DNA ligase), structural and packing (portal, major head subunit, major capsid protein, head-tail adaptor, tail completion protein, tail sheath protein, tail tube protein, tail tape measure protein, baseplate protein), and infectivity (anti-restriction, spanin, holin, endolysin). Two hypothetical proteins were identified as putative membrane proteins using TMHMM and Phobius. No detectable presence of antimicrobial resistance, virulence, or integrase-coding genes was observed.

Notably, the twelve identified structural proteins are localized closely together in the same region, suggesting that the handful of adjacent hypothetical proteins may also contribute to phage structure.

#### 3.2.2. Comparative Analysis

4HA13 has little sequence similarity to known phages. Its closest nucleotide homologues, *Escherichia* Mangalitsa and *Erwinia* phage Faunus, only had a percentage similarity (query coverage × % identity) of 50–53%, well below the proposed threshold for a new genus (70%) [50]. Comparative visualization of 4HA13 and five of its closest homologues, juxtaposed with its annotated genes, showed well-conserved regions (Figure 4). These regions mostly include genes involved in replication and packaging, such as RNA polymerase, DNA primase/helicase, DNA polymerase, and terminase large subunit. Also noted is the sequence conservation of the major capsid protein. 

**Figure 5:**
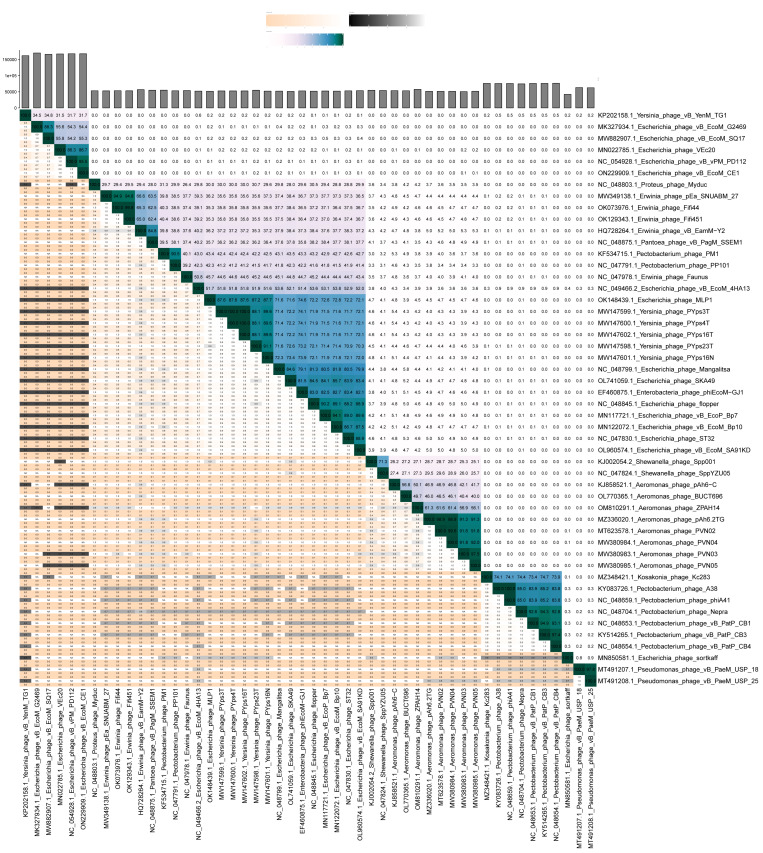
VIRIDIC heatmap of 4HA13 and its 50 closest nBlast homologues. The upper right half contains the intergenomic similarities between phage pairings, with intensity of color corresponding to level of similarity. The lower left half lists the percentage coverage of phage one, percentage alignment, and the percentage coverage of phage two.

**Figure 6:**
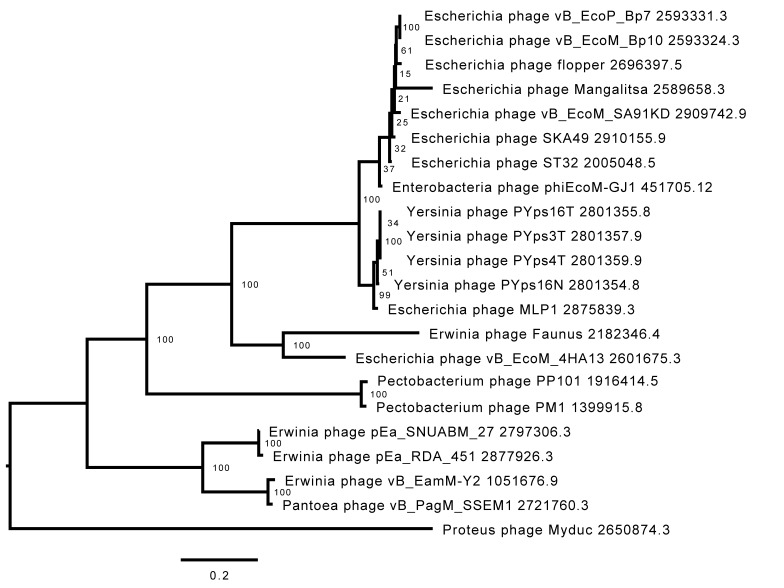
Phylogenetic tree of 4HA13 and its VIRIDIC clade members. The tree was constructed using PATRIC’s PGFams using 10 genes and an allocation of 3 genome exclusions.

VIRIDIC comparison of 4HA13 and its 49 closest nucleotide homologues showed distinct clades based on intergenomic similarities (Figure 5). 4HA13 is part of a large clade of 24 phages bordered by *Proteus* phage Myduc and *Escherichia* phage vB_EcoM_SA92KD, but does not belong to any of the subclades. Compared with *Erwinia* phage Faunus, it has an intergenomic similarity of 50.8% and complete alignment of 70% of both genomes. Due to the low similarities between the homologues, we constructed a phylogenetic tree using PATRIC global protein families (PGFams) of the 24 VIRIDC-identified relatives and it placed 4HA13 in a clade with *Erwinia* phage Faunus (Figure 6). 

Further comparative proteomics work carried out by Anany et al. identified 25 shared genes between 4HA13 and its homologues, which confirmed the five aforementioned genes (RNA polymerase, DNA polymerase/helicase, DNA polymerase, and terminase large subunit) [20]. Their phylogenetic tree built using concatenated amino acid sequence of the RNA polymerase and terminase large subunit largely supported the VIRIDIC clades. We were also able to show two large distinctly different clades, which they proposed as novel subfamilies—*Nefertitivirinae* and *Cleopatravirinae*. 4HA13 and its 24 VIRIDIC relatives belong to the latter, with 4HA13 representing a new genus (*Sabourvirus*).

### 3.3. Proteome Characterizations

**Figure 7:**
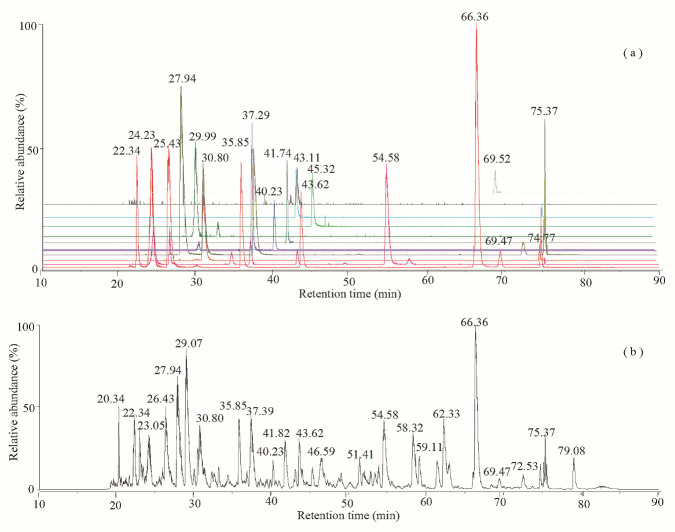
Ion chromatograms of the trypsin-treated 4HA13. Proteins were identified using LC MS/MS at a duration of 90 min. (**a**) Extracted ion chromatograms of the high abundance peptides of the top twelve proteins followed by the order of major capsid protein (gi|1735348871, red), tail tape measure protein (gi|1735348880, pink), tail sheath protein (gi|1735348876, orange), hypothetical protein AC4HA13_0057 (gi|1735348866, dark yellow), hypothetical protein AC4HA13_0041 (gi|1735348850, purple), tail tube initiator protein (gi|1735348882, blue), hypothetical protein AC4HA13_0070 (gi|1735348883, dark cyan), tail completion protein (gi|1735348875, oliver), baseplate wedge protein (gi|1735348886, green), tail tube protein (gi|1735348877, cyan), hypothetical protein AC4HA13_0076 (gi|1735348889, gray), and hypothetical protein AC4HA13_0049 (gi|1735348858, light gray). (**b**) Total ion chromatogram of a tryptic digest of 4HA13.

**Figure 8:**
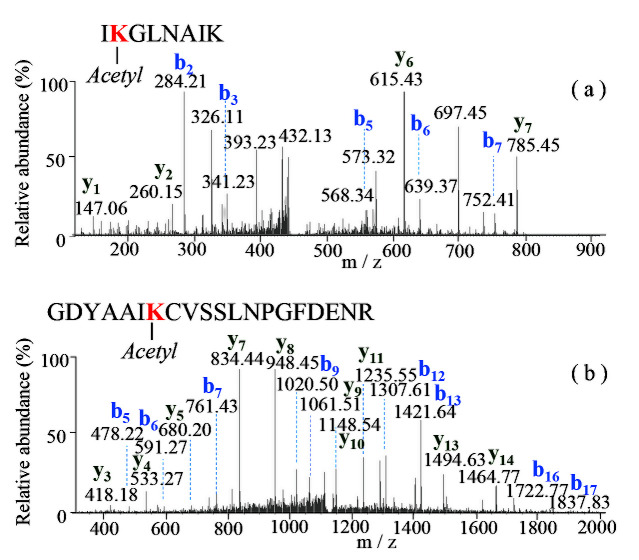
Identification of acetylated-lysine-containing peptides of 4HA13. (**a**) MS/MS spectrum of peptide 262–269 at *m/z* 449.7904 of a major capsid protein; (**b**) MS/MS spectrum of peptide 37–56 at *m/z* 1128.0223 of a tail completion protein. Symbols of b and y ions denote the fragments extended from the N-terminus and C-terminus of the peptides, respectively.

LC MS/MS analyses of the 4HA13 proteins followed by a Mascot data search identified 15 proteins with ≥50% sequence coverage including major capsid, head, tail structural, and non-structural components of the phage (Figure 7, Supplementary Table S2). Two common modifications, accompanied by the loss of methionine and acetylation, were found at the N-termini of several matured proteins. The loss of initiator methionine during the protein synthesis involves methionine aminopeptidase, which cleaves the N-terminal methionine adjacent to small amino acids (Ala, Pro, Ser, Thr) at the penultimate position [51]. N-terminal acetylation of proteins also occurs after the cleavage of initiating methionine, and the processing is catalyzed by N-terminal acetyltransferases [52], as observed in the proteins of tail tube initiator protein and baseplate wedge protein. In addition to the finding of protein N-terminal acetylation, the lysine acetylation was also identified in the two structural proteins of major capsid protein and tail completion protein. Tandem mass (MS/MS) spectra (Figure 8) of the Lys-containing tryptic peptides (262IKGLNAIK269 and 37GDYAAIKCVSSLNPGFDENR56) of the doubly charged ions at *m/z* 449.7904 and *m/z* 1128.0223 displayed a set of the N-terminal and C-terminal fragments, in which the mass increment of 42.010 Da of the only internal Lys residues (Lys263 of major capsid protein; Lys43 of a tail completion protein) indicated the modification by acetylation. The reversible acetylation enables lysine residues in the proteins that are resistant to trypsin digestion. It plays an important role in the epigenetic regulation and the phage cell signaling [53,54].

## 4. Conclusions

Non-O157 STEC continues to be a threat to food safety, increasing its reported incidence rate to surpass that of O157. Bacteriophages have several advantages over current chemical and physical microbial reduction approaches—their specificity, environmental friendliness, and little impact on the food’s organoleptic properties [55]. This study isolated and characterized *Escherichia* phage vB_EcoM-4HA13, which was highly specific for the non-motile *E. coli* O111 strain. 

Although 4HA13 is free of detectable integrase, antimicrobial resistance, or virulence-encoding genes, its narrow host range and poor adsorption kinetics do not bode well for its candidacy as a biocontrol agent. An ideal phage candidate for microbial reduction applications should have a wide enough host range to effectively reduce the pathogen, but narrow enough not to impact beneficial members of the bacterial community. It should also have rapid and effective adsorption, a short latent period, and a large burst size. The reason is twofold: (1) ease of high-titred preparation, and (2) fast and high reduction of the pathogen. Additional guidelines and considerations for an ideal phage candidate have been postulated in several works [56,57,58,59]. However, the 4HA13 phage can be used for developing a sensitive and highly specific detection approach for *E. coli* O111 strains after including more O111 strains in its host range study [60].

Based on comparative genomics, 4HA13 is suggested to be a novel species representing a new genus. Additional work carried out by Anany et al. on 4HA13 and its VIRIDIC clade relatives led to the proposal of a new phage family, *Chaseviridae,* and 4HA13 as the paradigm for the new genus *Sabourvirus* [20]. This is a case argument for sustained efforts towards phage isolation and characterization. Application potential aside, discoveries of new phages such as 4HA13 help further the phage community’s understanding of phage diversity, and contribute towards the modernization of the phage taxonomy system. 

## Data Availability

The genomic sequence of 4HA13 generated during this study is available at GenBank (accession number: MN136198.2). All other datasets generated during and/or analysed during the current study are available from the corresponding author upon reasonable request.

## Supplementary Materials

The following supporting information can be downloaded at: https://www.mdpi.com/article/10.3390/v14112356/s1, Table S1: *E. coli* strains and 4HA13 host range; Table S2: Protein identification of 4HA13 by tryptic digestion and LC MS/MS analyses.
