# Supplementary material for: Isolation, Characterization, and Genome Analysis of a Novel Bacteriophage, Escherichia Phage vB_EcoM-4HA13, Representing a New Phage Genus in the Novel Phage Family Chaseviridae
Source: Viruses. 2022 Oct 26;14(11):2356. doi: 10.3390/v14112356 (PMC9699118; doi:10.3390/v14112356)
Supplement: Supplementary file 1 [file viruses-14-02356-s001.zip › viruses-1966935-supplementary.pdf]

Supplementary Materials

Table S1. *E. coli* strains and 4HA13 Host Range.

	Serotype	AMR	Strain Number	Isolation Source	Source	Host Range	
						PHIDA	EOP
Generic *E. coli*	O2:H7	N/A	HA2018074	Human UTI	AAFC	N	0%
	O25:H4	N/A	HA2018075	Human UTI	AAFC	N	0%
	O28:NM	N/A	HA2018076	Human UTI	AAFC	N	0%
	O82:DM3Na	N/A	HA2018076	Unknown	AAFC	N	0%
	O114:H4	N/A	HA2018078	Human UTI	AAFC	N	0%
	DH5-α	N/A	HA2018027	Unknown	AAFC	D	0%
STEC	O26:H11	N/A	HA2018016	Bovine feces	PHAC-NML at Guelph	N	0%
	O26:H11	N/A	HA2018085	Human	PHAC-NML at Guelph	N	0%
	O45:H2	N/A	HA2018020	Bovine feces	PHAC-NML at Guelph	N	0%
	O103:H2	N/A	HA2018017	Bovine feces	PHAC-NML at Guelph	N	0%
	O103:H2	N/A	HA2018084	Human	PHAC-NML at Guelph	N	0%
	O111:NM	N/A	HA2018015	Bovine feces	PHAC-NML at Guelph	D+, 13 h	100%
	O111:H8	N/A	HA2018083	Bovine	PHAC-NML at Guelph	NL++, 18%	1%
	O121:H19	N/A	HA2018019	Human	PHAC-NML at Guelph	N	0%
	O145:NM	N/A	HA2018018	Bovine feces	PHAC-NML at Guelph	N	0%
	O157:H7	N/A	HA2018013		ATCC700927	N	0%
	O157:H7	N/A	HA2018079	Human UTI	AAFC	N	0%
Antimicrobial-resistant *E. coli*	N/A	AMP, CHL, SIX, STR, SXT, TET	HA2018155	Unknown	PHAC-NML at Guelph	N	0%
	N/A	AMP, CHL, CIP, GEN, NAL, SIX, SXT, TET,	HA2018160	Unknown	PHAC-NML at Guelph	N	0%
	N/A	AMP, CRO, GEN, STR, SIX, SXT, TET	HA2018163	Unknown	PHAC-NML at Guelph	N	0%
	N/A	AMC, AMP, FOX, CRO, CHL, GEN, SIX, STR, SXT, TET	HA2018164	Unknown	PHAC-NML at Guelph	N	0%

	N/A	TET	HA2018166	Unknown	PHAC-NML at Guelph	N	0%

*Antibiotics: AMC=amoxilcillin/clavulanic acid, AMP=Ampicillin, CHL=Chloramphenicol, CIP=ciprofloxacin, CRO=ceftriaxone, FOX=cefoxitin, GEN=gentamicin, NAL=nalidixic acid, SIX= sulfisoxazole, STR=streptomycin, SXT=trimethoprim/sulphamethoxazole, TET=tetracycline

C=completion inhibition of bacterial growth, D+=more than a 5-hour delay in initial detection of bacterial growth, D=less than a 5-hour delay, NL++=no delay on detection, but final optical density is less than 40% of control, N=no effect.

Table S2. Protein identification of 4HA13 by tryptic digestion and LC MS/MS analyses.

Protein name	NCBI accession number	Predicted mass	Sequence coverage (90 min LC run)	Sequence coverage (180 min LC run)
hypothetical protein AC4HA13_0057	gi|1735348866|gb|QEM43028.1|	16478	100%*@	100%*@
hypothetical protein AC4HA13_0070	gi|1735348883|gb|QEM43045.1|	13788	80%	80%
tail tube protein	gi|1735348877|gb|QEM43039.1|	16516	77%	76%
tail completion protein	gi|1735348875|gb|QEM43037.1|	19408	76%§	76%§
major capsid protein	gi|1735348871|gb|QEM43033.1|	37186	71%§	86%§
major head subunit precursor	gi|2062562666|gb|QEM43027.2|	38825	69%	61%
tail sheath protein	gi|1735348876|gb|QEM43038.1|	51283	66%	66%
tail tape measure protein	gi|1735348880|gb|QEM43042.1|	128217	64%	67%
single-stranded DNA-binding protein	gi|1735348822|gb|QEM42984.1|	13146	62%	62%
hypothetical protein AC4HA13_0060	gi|1735348872|gb|QEM43034.1|	16555	60%	61%
head-tail adaptor protein	gi|1735348874|gb|QEM43036.1|	13877	60%	60%
baseplate wedge protein	gi|1735348885|gb|QEM43047.1|	13468	58%*@	57%*@
hypothetical protein AC4HA13_0029	gi|1735348838|gb|QEM43000.1|	12922	53%	53%
hypothetical protein AC4HA13_0050	gi|1735348859|gb|QEM43021.1|	15743	52%	38%
hypothetical protein AC4HA13_0041	gi|1735348850|gb|QEM43012.1|	9546	50%	50%

* The N-terminal methionine are deleted in the protein sequences.

@ Acetylation at the N-terminus of the protein

§ Lysine acetylation of proteins: major capsid protein (peptide 262-269 at residues IKGLNAIK at m/z 449.7904:); tail completion protein (peptide 37-56 at residues GDYAAIKCVSSLNPGFDENR at m/z 1128.0223)
